# Role of cation structure in the phytotoxicity of ionic liquids: growth inhibition and oxidative stress in spring barley and common radish

**DOI:** 10.1007/s11356-017-9439-x

**Published:** 2017-06-22

**Authors:** Robert Biczak, Barbara Pawłowska, Arkadiusz Telesiński, Janusz Kapuśniak

**Affiliations:** 1 The Faculty of Mathematics and Natural Sciences, Jan Długosz University in Częstochowa, 13/15 Armii Krajowej Av, 42-200 Częstochowa, Poland; 2 The Faculty of Environmental Management and Agriculture, West Pomeranian University of Technology, Juliusza Słowackiego st. 17, 71-434 Szczecin, Poland

**Keywords:** Ionic liquids, Phytotoxicity, Oxidative stress, Antioxidant enzyme activity, Photosynthetic pigments

## Abstract

The present study determines the influence of three ionic liquids (ILs) containing cations with diversified structure on the growth and development of spring barley seedlings and common radish leaves. Increasing amounts of 1-butyl-1-methylpyrrolidinium hexafluorophosphate [Pyrrol][PF_6_], 1-butyl-1-methylpiperidinium hexafluorophosphate [Piper][PF_6_], and 1-butyl-4-methylpyridinium hexafluorophosphate [Pyrid][PF_6_] were added to the soil on which both plants were cultivated. The results of this studies showed that the applied ILs were highly toxic for plants, demonstrated by the inhibition of length of plant shoots and roots, decrease of fresh mass, and increase of dry weight content. Common radish turned out to be the plant with higher resistance to the used ILs. The differences in the cation structure did not influence phytotoxity of ILs for spring barley. Furthermore, all ILs led to a decrease of photosynthetic pigments, which was directly followed by decreased primary production in plants. Oxidative stress in plants occurred due to the presence of ILs in the soil, which was demonstrated by the increase of malondialdehyde (MDA) content, changes in the H_2_O_2_ level, and antioxidant enzymes such as superoxide dismutase (SOD), catalase (CAT), and peroxidase (POD). The changes in the chlorophyll contents and the increase of POD activity turned out to be the most significant oxidative stress biomarkers in spring barley and common radish. Both spring barley and radish exposed to ILs accumulated a large amount of fluoride ion.

**Electronic supplementary material:**

The online version of this article (doi:10.1007/s11356-017-9439-x) contains supplementary material, which is available to authorized users.

## Introduction

In the last decade, interest on the so-called clean technologies in chemical industry has been increased due to technological and environmental reasons. It gives rise to a continuous search for alternatives to the conventional organic solvents. Ionic liquids may become such an alternative. Their basic features, such as low melting temperatures, low vapor pressure, polarity, high thermal and electrochemical stability, high ionic conductivity, and non-flammability, prevent solvent loss and increase the safety for persons employed in the processes of industrial chemical synthesis. However, the study conducted on the influence of ionic liquids (ILs) on the aquatic and soil environment gives rise to a serious concern about the possibility of a permanent contamination of these ecosystems with the discussed compounds. IL toxicity has been observed in microorganisms, fungi, algae, terrestrial plants, invertebrates, and vertebrates (Pham et al. 2010; Petkovic et al. 2012; Peric et al. 2013; Cvjetko Bubalo et al. 2014b; Feder-Kubis and Tomczuk 2013; Messali et al. 2013).

When large amounts of ILs become available in the market and are used in numerous processes, it should be borne in mind that they will penetrate into the soil environment, where the phenomenon of soil sorption related to the presence of humus and inorganic colloids may be limited in the top layer of the soil, close to the roots, posing direct threat to plants. Therefore, numerous publications have been published, evaluating the level of ILs’ influence on the growth and development of terrestrial plants (Biczak et al. 2010, 2013, 2014a, 2015; Matzke et al. 2008a, b; Studzińska and Buszewski 2009). In the abovementioned papers, ILs’ phytotoxicity was determined primarily on the basis of plant growth inhibition, but as mentioned by Cvjetko Bubalo et al. (2014a), the IL toxicity mechanism has not yet been fully understood. Therefore, a view gains increasing popularity in scientific studies in which ILs’ phytotoxicity is related to the generation of oxidative stress in plants by these compounds (Liu et al. 2013, 2014, 2015a, b, 2016a, b; Biczak 2016; Biczak et al. 2016a, 2016b; Pawłowska and Biczak 2016).

An opinion prevails in the scientific literature in which the IL phytotoxicity largely depends on the cation and the length of substituent (Cvjetko Bubalo et al. 2014b; Biczak et al. 2014a; Matzke et al. 2008a, b; Studzińska and Buszewski 2009), while to a lesser extent, it depends on the anion type (Cvjetko Bubalo et al. 2014b; Studzińska and Buszewski 2009; Liu et al. 2016a, b; Biczak et al. 2014b). However, scientific reports on the correlation of IL phytotoxicity and their cations are limited. Only Pham et al. (2016) demonstrated the higher toxicity of ILs with pyridinium cations compared to pyrrolidinium and imidazolium cations in *Pseudokirchneriella subcapitata* algae. Therefore, the objective of the present study was to determine and compare the toxic effects of ILs with different cation structures to spring barley and common radish. The study focused on relatively popular ILs, which are used in chemical synthesis, electrochemistry, and biotechnological processes, containing hexafluorophosphate anion and the following cations: 1-butyl-1-methylpyrrolidinium, 1-butyl-1-methylpiperidinium, and 1-butyl-4-methylpyridinium (Bae et al. 2013; Atta et al. 2016; Elgharbawy et al. 2016). In order to compare the phytotoxicity of these ILs and at the same time demonstrate the effects of cations on IL toxicity, apart from traditional phytotoxicity biomarkers including shoot length, root length, and fresh and dry weight yield, the present study also examined the oxidative stress indicators in the seedlings of spring barley and radish such as the malondialdehyde (MDA) content, H_2_O_2_, photosynthetic pigments, and activity of superoxide dismutase (SOD), peroxidase (POD), and catalase (CAT). Since the used ILs contain PF_6_^−^ anions, the effect of fluoride ions was evaluated for both spring barley and common radish as fluoroacetate, a harmful compound to plants, may be formed when fluoride is absorbed from the soil (Baunthiyal and Pandey 2012). The choice of spring barley for the study was dictated by the evidence that it is the fourth most common cereal species in production and acreage, and radish is a popular vegetable, enriching the human diet with a number of microelements and macroelements and vitamins (Schubert and Jahren 2011; Dragišić Maksimović et al. 2013; Arias-Baldrich et al. 2015).

## Materials and methods

### Ionic liquids

**Fig. 1:**
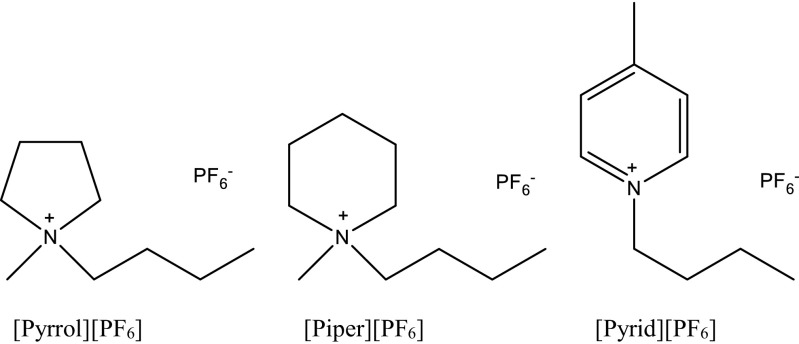
Structures of the analyzed ILs

All ILs used in these studies were purchased from Sigma-Aldrich Chemical Co. Chemical structures and abbreviations of ILs 1-butyl-1-methylpyrolidinium hexafluorophosphate (≥97.5% purity), 1-butyl-1-methylpiperidinium hexafluorophosphate (≥97.5% purity), and 1-butyl-4-methylpyridinium hexafluorophosphate (≥97.0% purity) are presented in Fig. 1.

### Evaluation of ILs’ phytotoxicity

Determination of phytotoxicity of ILs with different cation structures was performed according to the OECD/OCDE guidelines (2006). The following concentrations of ILs were used: 0, 1, 10, 50, 100, 400, 700, and 1000 mg of compound per 1 kg of dry weight (DW) of the soil. When preparing the abovementioned concentrations, appropriate amount of ILs was dissolved in acetone and subsequently mixed with quartz sand. After overhead evaporation of acetone, quartz sand containing ILs was mixed with the soil. Three independent samples were prepared for each concentration of IL.

Following the above procedure, control samples were prepared by adding acetone to the sand, but without ILs. In this study, loam was used as soil. It contained about 11% of fraction with a diameter of <0.02 mm and organic carbon of 9.5 g kg^−1^. The pH was 6.1. Plastic pots were filled with the prepared medium; thereafter, 20 seeds of spring barley (*Hordeum vulgare*) and common radish (*Raphanus sativus* L. *radicula* Pers.) derived from the same source were added. Seed germination and seedlings’ growth (14 days) were carried out under strictly controlled conditions: soil moisture, 70% ppw; temperature, 20 ± 2 °C; and constant illumination, 170 μmol m^−2^ s^−1^ for 16-h day/8-h night. The experiments were conducted in a vegetation hall, which belongs to the Department of Biochemistry and Ecotoxicology at Jan Długosz University in Częstochowa.

Phytotoxicity of the studied ILs for spring barley and common radish was estimated based on, among others, the yield of fresh weight of seedlings, dry weight content, and length of the shoots and roots. The inhibition factor of fresh weight and lengths of shoots and roots were calculated according to the study published by Wang et al. (2009). Using non-linear regression analysis, EC_50_ was estimated based on the calculated inhibition, with the GraphPad Prism software (GraphPad Software, Inc., La Jolla, CA, USA). Furthermore, both germination potential (GP) and germination rate (GR) of spring barley and common radish seeds were determined. The seeds with longer than 2-mm germ were considered germinated (Liu et al. 2014).

In fresh plant material (spring barley seedlings and common radish leaves), the content of all assimilation pigments, malondialdehyde, H_2_O_2_, and enzymes’ activity was determined. In the dried material, the analysis of total fluorine content was performed. Samples treated with high concentrations of ILs were not included in some analyses because the growth inhibition of spring barley and radish shoots was extremely strong at these concentrations.

### Determination of total fluoride content

Based on the method described by Eyde (1982), the total fluoride content in spring barley seedlings and common radish leaves was assessed. Dried and ground samples were fused in nickel crucibles with sodium hydroxide. Water, diluted hydrochloric acid, and citrate buffer solution were added to the melt. Fluoride concentration was determined in the presence of TISAB III buffer using the potentiometric method with an Orion Research ion-selective electrode. The total fluoride content was expressed in mg kg^−1^ of DW.

### Determination of assimilation pigment content

According to the methodology presented by Oren et al. (1993), the content of assimilation pigments was determined by spectrophotometry. With the addition of 80% acetone solution cooled to 4 °C, weighed portion (200 mg) of fresh spring barley seedlings and common radish leaves was homogenized. The resulting extract was transferred into centrifuge tubes and left in the dark for 24 h. The extract was then centrifuged, and in the filtrate, the content of assimilation dyes was determined by measuring the absorbance at 470, 647, and 664 nm. The contents of chlorophylls and carotenoids were expressed in mg g^−1^ of dry plant weight (DW).

### Determination of malondialdehyde and hydrogen peroxide (H_2_O_2_)

With the addition of 0.1% trichloroacetic acid solution cooled to 4 °C, 500 mg of fresh spring barley seedlings and common radish leaves were homogenized. After centrifugation, MDA and H_2_O_2_ content were determined in the obtained supernatant according to the procedures described by Hodges et al. (1999) and Singh et al. (2007), respectively. As a substrate to determine MDA, thiobarbituric acid was used and the MDA content was determined by measuring the absorbance at 532 and 600 nm. In order to determine the content of H_2_O_2_, the absorbance was measured at a wavelength of 390 nm for the reaction mixture consisting of supernatant, phosphate buffer (pH 7.0), and potassium iodide. The contents of MDA and H_2_O_2_ were calculated using the extinction coefficient equaling 155 mM^−1^ cm^−1^ and expressed in μmol g^−1^ fresh weight (FW).

### Determination of superoxide dismutase, catalase, and peroxidase activity

For the determination of antioxidant enzyme activity, enzymatic extracts were obtained by homogenizing the weighed portion (500 mg) of fresh spring barley seedlings and common radish leaves with the addition of cooled (4 °C) extraction mixture. The mixture contained phosphate buffer (pH 7.4), 1 mM EDTA solution, and 0.1% polyvinylpyrrolidone solution. After centrifugation, the obtained supernatant was also used to determine the protein content.

SOD [EC 1.15.1.1] activity was determined spectrophotometrically by measuring the degree of reduction of nitroblue tetrazolium (NBT) by superoxide anion formed as a result of photochemical reduction of riboflavin in the presence of light (Giannopolitis and Ries 1977). NBT reduction is inhibited by SOD. For determining the activity of SOD, measurement of absorbance of reaction mixture at a wavelength of 560 nm was performed. SOD activity was expressed as U mg^−1^ protein, where 1 U is the amount of enzyme that induces 50% inhibition of NBT reduction.

CAT activity [EC 1.11.1.6] was determined by the decomposition of H_2_O_2_ by this enzyme during 15 min, and H_2_O_2_ remaining in the reaction mixture was titrated with 0.01 N KMnO_4_ (Kar and Mishra 1976). CAT activity was expressed as U mg^−1^ protein min^−1^.

The activity of POD [1.11.1.7] was determined spectrophotometrically by the rate of guaiacol oxidation in the presence of H_2_O_2_ via enzyme occurring in a given volume of extract during 1 min (Abassi et al. 1998). Absorbance measurement of the reaction mixture at a wavelength of 470 nm was performed, in order to measure the activity of POD. POD activity was expressed as U mg^−1^ protein min^−1^.

The total protein content necessary to calculate the activity of SOD, CAT, and POD was determined by the Bradford (1976) assay.

### Statistical analysis

Using two-way analysis of variance, results were subjected to statistical analysis. Tukey’s test with *p* < 0.05 was used to determine the significance of differences. Data present in tables and figures are expressed as means ± standard deviation obtained from three measurement replicates.

## Results and discussion

### Phytotoxicity assay

**Fig. 2:**
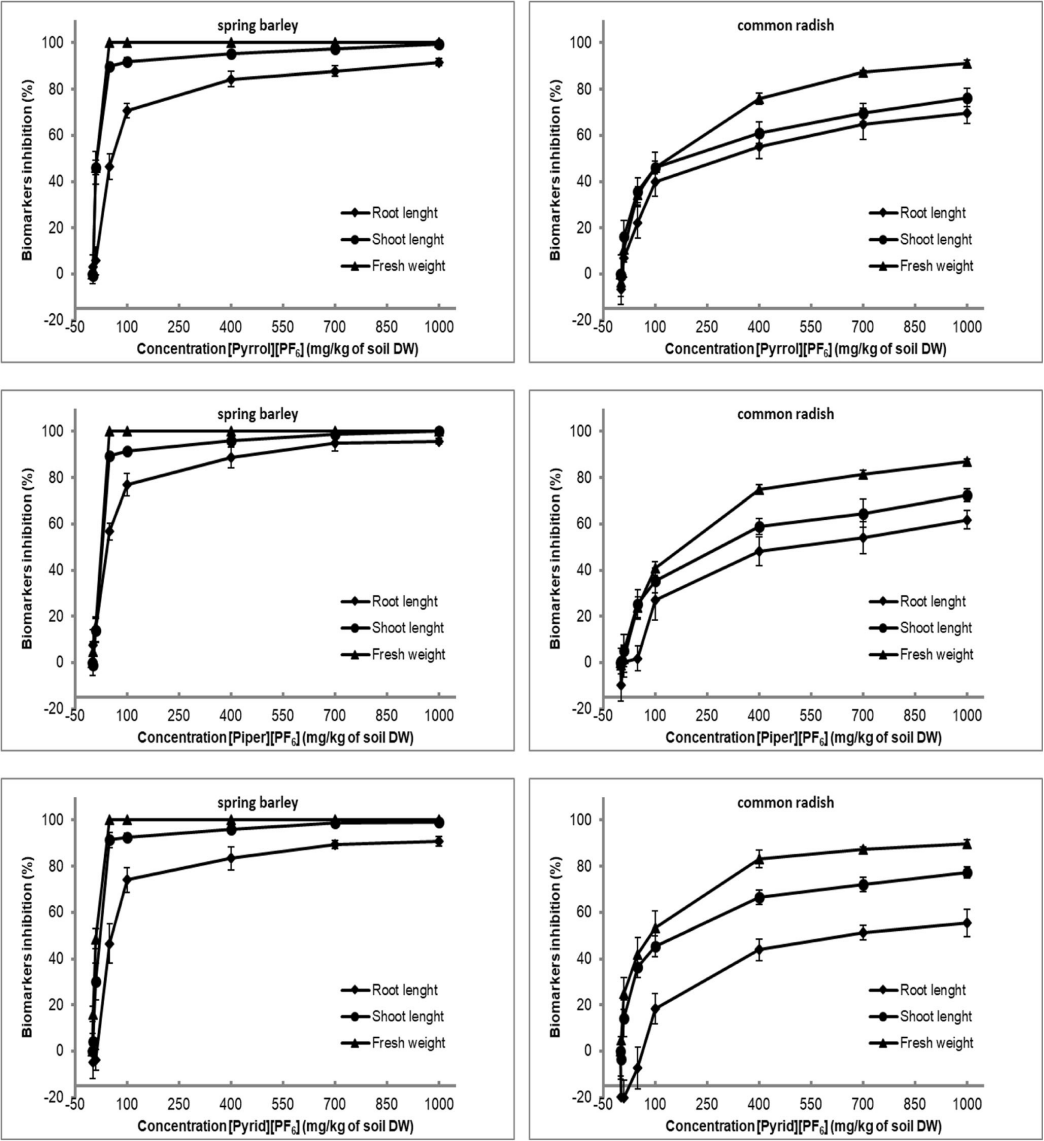
The inhibition rate (%) for shoot length, root length, and fresh weight of spring barley and common radish after exposure to ILs

The analysis of the obtained results on the influence of the used ILs on the growth and development of spring barley and common radish revealed that all the tested compounds exhibited phytotoxic effect. This is demonstrated by the decrease of seedlings’ fresh weight yield, decrease of the length of their shoots and roots, and the calculated EC_50_ values on the basis of inhibition of these toxicity biomarkers (Fig. 2, Table 1).

**Table 1:** The EC_50_ values and 95% confidence intervals for spring barley and common radish following exposure to ILs

Biomarkers	[Pyrrol][PF_6_]		[Piper][PF_6_]		[Pyrid][PF_6_]	
	EC_50_ (mg kg^−1^ of soil DW)	95% Confidence intervals	EC_50_ (mg kg^−1^ of soil DW)	95% Confidence intervals	EC_50_ (mg kg^−1^ of soil DW)	95% Confidence intervals
Spring barley						
Root length	88.97	71.24–111.10	114.9	74.64–177.00	97.78	69.63–137.30
Shoot length	10.16	7.96–12.98	19.86	14.14–27.88	14.49	12.08–19.87
Fresh weight	10.04	7.55–11.67	11.40	8.66–16.68	10.31	9.01–12.10
Common radish						
Root length	137.20	72.07–156.10	120.40	28.64–255.80	101.40	67.41–152.70
Shoot length	228.50	102.80–387.20	149.60	35.51–300.60	90.87	50.92–162.10
Fresh weight	172.00	84.83–248.70	115.20	79.07–168.00	129.60	82.20–204.20

The observed phytotoxicity was significantly influenced by ILs’ concentrations. This is confirmed by the reports of other authors, who also point out to the strict dependency of the level of phytotoxicity on the used ILs’ concentration. Numerous papers (Biczak et al. 2010, 2013, 2014a, 2015; Cvjetko Bubalo et al. 2014a; Liu et al. 2013, 2014, 2015a, b; Biczak 2016; Pawłowska and Biczak 2016; Wang et al. 2009) demonstrate the linear relationship between phytotoxicity of quaternary ammonium salts (QASs) and ILs and the concentration of the compound in soil. Such strong correlations are observed mainly for the high concentrations of these chemicals, whereas ILs used in low concentrations may have a stimulatory effect on the growth and development of plants (Liu et al. 2013, 2015b, 2016b).

Spring barley was very sensitive to the used ILs, and the plant material could only be obtained in the case of ILs’ concentrations of 1 and 10 mg kg^−1^ of the soil DW. Common radish exhibited higher resistance to the tested ILs (Fig. 2, Table 1). The difference in the sensitivity of spring barley and common radish observed in the present study may stem from the reaction of the root systems of these plants to the tested salts. At higher IL concentrations (50–1000 mg kg^−1^ of soil DW), over 90% spring barley root growth inhibition was observed, which effectively disabled the plants to absorb and transport water and nutrients. As reported by Chapman et al. (2012), proper root development determined the optimum growth and development of each plant. Such root inhibition was not observed for common radish.

It is shown that there is no significant effect of cation structures on the phytotoxicity of the tested ILs to spring barley and common radish. The results of shoot length, root length, fresh weight, and EC_50_ values of both plants showed no remarkable differences (Fig. 2, Table 1). This is further illustrated by the photographs of the seedlings cultivated on the soil with increasing ILs’ concentration, presented in Supplementary Materials (Suppl.Figs 1 and 2). Currently, the obtained results cannot be compared to the other studies describing the IL effect on terrestrial plants. Only Pham et al. (2016) compared the effect of pyridinium, pyrolidinium, and imidazolium bromides on the growth of the *P. subcapitata* algae and stated that the IL with the pyridinium cation was more toxic to the algae than the other compounds. Cho et al. (2008) evaluated toxicity of imidazolium, pyridinium, pyrrolidinium, phosphonium, and ammonium ILs on the algae, *Selenastrum capricornutum*, and the obtained results showed that the pyrrolidinium cation was the least toxic. However, Stolte et al. (2007) proved higher toxicity of ILs containing aromatic cation for duckweed (*Lemma minor*) and microorganisms. The study carried out by Couling et al. (2006) pointed at the fact that the higher amount of nitrogen atoms in a cation ring caused the higher toxicity of ionic liquids for aquatic organisms. However, these studies were conducted in an aquatic environment, whereas our study was conducted in the soil, which is a very complex environment for toxicity study. The obtained results can be influenced by numerous factors, such as pH of the soil, content of humus substances, soil colloids, and sorption level (Cvjetko Bubalo et al. 2014b; Matzke et al. 2008a, 2009; Mrozik et al. 2009; Studzińska et al. 2009).

Moreover, the present study determined the influence of ILs with different cation on the dry weight content in spring barley seedlings and common radish leaves. In both plants, an increase of dry weight was observed, correlated to the increase of ILs’ concentration in the soil (Table 2).

**Table 2:** Effect of ILs on the dry weight (g g^−1^ FW) in spring barley seedlings and common radish leaves

Dry weight content (g g^−1^ FW) in spring barley seedlings and common radish leaves			
IL concentration (mg kg^−1^ of soil DW)	[Pyrrol][PF_6_]	[Piper][PF_6_]	[Pyrid][PF_6_]
Spring barley			
0	0.0787 ± 0.0013c	0.0757 ± 0.0032c	0.0763 ± 0.0014c
1	0.0793 ± 0.0041c	0.0762 ± 0.0019c	0.0760 ± 0.0008c
10	0.1047 ± 0.0049a	0.0868 ± 0.0020ba	0.0971 ± 0.0105ab
50	–	–	–
100	–	–	–
400	–	–	–
700	–	–	–
1000	–	–	–
Common radish			
0	0.0609 ± 0.0025ijkl	0.0668 ± 0.0122hijk	0.0508 ± 0.0005kl
1	0.0533 ± 0.0017kl	0.0594 ± 0.0043jkl	0.0494 ± 0.0018l
10	0.0622 ± 0.0041ijkl	0.0594 ± 0.0047jkl	0.0541 ± 0.0024kl
50	0.0765 ± 0.0064hc	0.0720 ± 0.0010hc	0.0719 ± 0.0057hij
100	0.0952 ± 0.0090g	0.0766 ± 0.0040g	0.0804 ± 0.0057gh
400	0.1786 ± 0.0033f	0.1748 ± 0.0130f	0.2052 ± 0.0037dc
700	0.2444 ± 0.0010c	0.2045 ± 0.0066c	0.2209 ± 0.0051d
1000	0.2596 ± 0.0045bc	0.2878 ± 0.0012a	0.2675 ± 0.0101b

Data are expressed as a mean ± SD of three replicates for each concentration. Values denoted by the same letters in the columns do not differ statistically at *p* < 0.05

High ionic liquid concentrations lead to soil salinity, which, in turn, disturbs the water metabolism in plants, leading to the observed increased level of dry weight in both experimental plants. Biczak et al. (2013, 2014a, 2015, 2016b), Biczak (2016), Matusiak et al. (2013), and Pawłowska and Biczak (2016) came to analogous conclusions in their studies on the determination of toxicity of different chemicals for terrestrial plants.

Increase in the concentration of [Pyrrol][PF_6_], [Piper][PF_6_], and [Piryd][PF_6_] in the soil led to a systematic decrease of GP and GR values of spring barley seeds. In the case of common radish grown on the soil with the addition of ILs, a small decrease in the germination potential and germination rate of this plant seeds was noted, especially after the application of the highest concentrations of [Pyrrol][PF_6_] (Suppl.Table 1). Liu et al. (2014) described a similar decrease in germination capacity of wheat and spring barley seeds under the influence of imidazolium ILs.

### Effect of ILs on fluoride content

In the discussed studies, the content of fluoride ions in seedlings of spring barley and radish leaves was determined (Table 3).

**Table 3:** Effect of ILs on the fluoride (mg kg^−1^ DW) content in spring barley seedlings and common radish leaves

The fluoride content (mg kg^−1^ FW) in spring barley seedlings and common radish leaves			
IL concentration (mg kg^−1^ of soil DW)	[Pyrrol][PF_6_]	[Piper][PF_6_]	[Pyrid][PF_6_]
Spring barley			
0	4.306 ± 0.120a	4.338 ± 0.037a	4.279 ± 0.228a
1	4.359 ± 0.170a	4.369 ± 0.076a	4.351 ± 0.151a
10	4.287 ± 0.090a	4.396 ± 0.220a	4.477 ± 0.175a
50	–	–	–
100	–	–	–
400	–	–	–
700	–	–	–
1000	–	–	–
Common radish			
0	2.094 ± 0.130g	2.073 ± 0.052g	2.109 ± 0.090f
1	2.132 ± 0.158g	2.102 ± 0.100g	2.279 ± 0.165f
10	2.699 ± 0.306fg	3.274 ± 0.240f	2.731 ± 0.077fg
50	5.253 ± 0.207e	5.863 ± 0.126e	5.140 ± 0.129e
100	7.671 ± 0.211d	8.010 ± 0.144d	7.616 ± 0.012d
400	9.510 ± 0.179c	9.805 ± 0.452c	9.218 ± 0.096c
700	12.106 ± 0.172b	12.298 ± 0.678b	12.104 ± 0.198b
1000	14.386 ± 0.318a	15.103 ± 0.396a	14.584 ± 0.256a

Data are expressed as a mean ± SD of three replicates for each concentration. Values denoted by the same letters in the columns do not differ statistically at *p* < 0.05

Common radish accumulated large amounts of fluoride ions in its leaves, and the observed increase in the level of these anions was directly proportional to the increase in concentration of all ILs in the soil. In case of application of all studied ILs in a dosage of 1000 mg kg^−1^ of soil DW, the amount of fluoride in plants was approximately seven times higher than in the control groups. Such changes were not established for spring barley, probably because the analyses were possible to conduct only for the objects with the concentration of 10 mg ILs per 1 kg of soil DW. The effect of toxic fluoride on plants is visible in the form of chlorosis, peripheral necrosis, leaf distortion and malformation, and abnormal fruit development (Pandey et al. 2014). Telesiński and Śnioszek (2009) determined that fluorine has a negative influence on the assimilation and photosynthesis processes in plants. These phenomena stem from the destructive influence of F^−^ on chloroplasts.

### Effect of ILs on pigment content

An analysis of the results on the effect of ILs on photosynthetic pigments’ level in spring barley seedlings and common radish leaves demonstrates the inhibitory influence of these salts on the level of chlorophylls and carotenoids. All the ILs used in the experiment led to a systematic decrease in the content of photosynthetic pigments in both plants, which was correlated to the increase of these substances in the soil (Table 4).

**Table 4:** Effect of ILs on photosynthetic pigment content (mg g^−1^ DW) in spring barley seedlings and common radish leaves

Concentration of ILs (mg kg^−1^ of soil DW)		Pigments (mg g^−1^ DW)					
		Chl*a*	Chl*b*	Car	Chl*a* + Chl*b*	Chl*a*/Chl*b*	Chl(*a* + *b*)/Car
Spring barley							
[Pyrrol][PF_6_]	0	13.196 ± 0.341de	3.520 ± 0.042c	3.189 ± 0.058de	16.716 ± 0.377d	3.749 ± 0.064a	5.242 ± 0.024b
	1	13.923 ± 0.665bcd	3.498 ± 0.194c	3.486 ± 0.115b	17.420 ± 0.512cd	3.995 ± 0.409a	4.998 ± 0.025e
	10	10.374 ± 0.157f	2.684 ± 0.005e	2.568 ± 0.035f	13.058 ± 0.156f	3.865 ± 0.059a	5.086 ± 0.009cdc
	50	–	–	–	–	–	–
	100	–	–	–	–	–	–
	400	–	–	–	–	–	–
	700	–	–	–	–	–	–
	1000	–	–	–	–	–	–
[Piper][PF_6_]	0	15.092 ± 0.110a	3.989 ± 0.051a	3.461 ± 0.011b	19.201 ± 0.147a	3.814 ± 0.038a	5.548 ± 0.025a
	1	14.381 ± 0.017b	3.847 ± 0.013ab	3.463 ± 0.015b	18.228 ± 0.027b	3.738 ± 0.010a	5.264 ± 0.015b
	10	13.522 ± 0.283cd	3.626 ± 0.051bc	3.294 ± 0.058cd	17.148 ± 0.329cd	3.729 ± 0.038a	5.205 ± 0.018bc
	50	–	–	–	–	–	–
	100	–	–	–	–	–	–
	400	–	–	–	–	–	–
	700	–	–	–	–	–	–
	1000	–	–	–	–	–	–
[Pyrid][PF_6_]	0	15.420 ± 0.094a	4.055 ± 0.014a	3.942 ± 0.057a	19.476 ± 0.082a	3.803 ± 0.035a	4.941 ± 0.087e
	1	14.018 ± 0.151bc	3.654 ± 0.083bc	3.426 ± 0.033bc	17.672 ± 0.223bc	3.837 ± 0.060a	5.158 ± 0.112bcd
	10	12.547 ± 0.102e	3.116 ± 0.051d	3.115 ± 0.005e	15.663 ± 0.124e	4.027 ± 0.067a	5.028 ± 0.045de
	50	–	–	–	–	–	–
	100	–	–	–	–	–	–
	400	–	–	–	–	–	–
	700	–	–	–	–	–	–
	1000	–	–	–	–	–	–
Common radish							
[Pyrrol][PF_6_]	0	11.732 ± 0.065a	4.001 ± 0.027ab	3.193 ± 0.081a	15.732 ± 0.090a	2.932 ± 0.010def	4.930 ± 0.151abcde
	1	9.012 ± 0.294def	3.118 ± 0.093de	2.374 ± 0.128cde	12.130 ± 0.385ef	2.890 ± 0.021ef	5.116 ± 0.180abcde
	10	8.702 ± 0.029fg	2.773 ± 0.031fg	2.338 ± 0.013def	11.475 ± 0.047gh	3.139 ± 0.033cdef	4.909 ± 0.032abcdef
	50	7.521 ± 0.027i	2.570 ± 0.092g	2.011 ± 0.163fg	10.091 ± 0.108i	2.929 ± 0.102def	5.038 ± 0.384abcde
	100	7.665 ± 0.120i	2.642 ± 0.030g	1.934 ± 0.015g	10.308 ± 0.094i	2.902 ± 0.076ef	5.331 ± 0.066abc
	400	5.051 ± 0.119j	1.598 ± 0.047h	1.302 ± 0.030h	6.648 ± 0.164j	3.162 ± 0.033cdef	5.106 ± 0.060abcde
	700	2.187 ± 0.145m	0.595 ± 0.078i	0.641 ± 0.025jk	2.782 ± 0.067m	3.745 ± 0.192bcdef	4.343 ± 0.270efg
	1000	1.737 ± 0.068no	0.401 ± 0.026j	0.522 ± 0.006k	2.137 ± 0.045no	4.356 ± 0.462bcd	4.096 ± 0.120fg
[Piper][PF_6_]	0	10.069 ± 0.039c	3.282 ± 0.029cde	2.591 ± 0.016cd	13.351 ± 0.062d	3.068 ± 0.022cdef	5.153 ± 0.019abcde
	1	10.589 ± 0.260b	3.502 ± 0.058c	2.716 ± 0.068bc	14.091 ± 0.317c	3.033 ± 0.026cdef	5.188 ± 0.016abcde
	10	10.174 ± 0.028c	3.335 ± 0.011cd	2.613 ± 0.005cd	13.508 ± 0.017d	3.051 ± 0.019cdef	5.171 ± 0.009abcde
	50	9.253 ± 0.079de	2.600 ± 0.016a	2.147 ± 0.004efg	11.854 ± 0.064fg	3.559 ± 0.052bcdef	5.520 ± 0.023ab
	100	8.908 ± 0.152ef	2.550 ± 0.003g	2.172 ± 0.037efg	11.458 ± 0.155gh	3.493 ± 0.055bcdef	5.276 ± 0.029abcd
	400	4.850 ± 0.209j	1.170 ± 0.044i	1.243 ± 0.049hi	6.020 ± 0.253k	4.145 ± 0.025bcdef	4.842 ± 0.013abcdef
	700	3.909 ± 0.079k	0.929 ± 0.019i	1.056 ± 0.022hi	4.838 ± 0.096l	4.208 ± 0.048bcde	4.581 ± 0.013cdefg
	1000	2.064 ± 0.027mn	0.330 ± 0.088j	0.626 ± 0.079jk	2.394 ± 0.075mno	6.589 ± 0.922a	3.871 ± 0.537g
[Pyrid][PF_6_]	0	11.601 ± 0.113a	4.140 ± 0.149a	3.161 ± 0.066a	15.741 ± 0.258a	2.804 ± 0.075ef	4.982 ± 0.183abcde
	1	10.893 ± 0.083b	3.813 ± 0.121b	3.013 ± 0.075ab	14.705 ± 0.168b	2.859 ± 0.077ef	4.882 ± 0.140abcde
	10	9.337 ± 0.018d	3.121 ± 0.155de	2.480 ± 0.078cde	12.459 ± 0.167e	2.996 ± 0.142cdef	5.028 ± 0.229abcde
	50	8.313 ± 0.022gh	3.018 ± 0.256ef	2.267 ± 0.460defg	11.331 ± 0.263gh	2.768 ± 0.233f	5.113 ± 0.849abcde
	100	8.222 ± 0.222h	2.778 ± 0.071fg	1.943 ± 0.025g	11.001 ± 0.217h	2.961 ± 0.120def	5.663 ± 0.163a
	400	3.330 ± 0.126l	0.989 ± 0.054i	0.913 ± 0.056ij	4.319 ± 0.180l	3.369 ± 0.059bcdef	4.747 ± 0.399bcdef
	700	2.139 ± 0.050m	0.489 ± 0.047j	0.548 ± 0.013jk	2.629 ± 0.003mn	4.403 ± 0.500bc	4.504 ± 0.095cdefg
	1000	1.612 ± 0.021o	0.336 ± 0.008j	0.441 ± 0.048k	1.948 ± 0.015o	4.796 ± 0.177b	4.452 ± 0.491defg

Data are expressed as a mean ± SD of three replicates for each concentration. Values denoted by the same letters in the columns do not differ statistically at *p* < 0.05

A similar decrease in the photosynthetic pigment content in spring barley and wheat seedlings and radish and broad bean leaves, duckweed, and algae under IL influence was also observed by Cvjetko Bubalo et al. (2014a), Biczak (2016), Biczak et al. (2016a), Pawłowska and Biczak (2016), Wang et al. (2009), Liu et al. (2014), Zhang et al. (2013), and Ma et al. (2010). The cited authors demonstrate that the presence of ILs in the environment generates a high level of oxidative stress in plants, which is linked to the elevated reactive oxygen species (ROS) production (Anjaneyulu et al. 2014; Di Baccio et al. 2014; Oukarroum et al. 2015). Some authors (Cvjetko Bubalo et al. 2014a; Biczak 2016; Biczak et al. 2016a, b) also report that chlorophyll content constitutes the most important biomarker of oxidative stress because its changes are directly correlated with the inhibition of the growth and yield of plants. Islam et al. (2014) and Herman et al. (1998) believe that chlorophyll level reflects the health of plant leaves.

Besides the content of photosynthetic pigments, the following indexes are also used to evaluate the physiological changes: Chl*a*/Chl*b* ratio and Chl(*a + b*)/Car ratio. The increase of Chl*a*/Chl*b* value and decrease of Chl(*a + b*)/Car value indicate the occurrence of oxidative stress in plants (Arias-Baldrich et al. 2015; Chen et al. 2014). In addition, the decrease of Chl(*a + b*)/Car value indicates antioxidant defense of the plant organism, by increasing carotenoid content. Carotenoids are efficient ROS scavengers, thus protecting PSI and PSII photosystems (Arias-Baldrich et al. 2015; Wang et al. 2009; Chen et al. 2014; Gengmao et al. 2015). No statistically significant differences in the values of both mentioned indexes were determined in the spring barley seedlings. In the case of common radish, value of the Chl*a*/Chl*b* index increased on the samples with the highest ILs’ concentration; additionally, for the same samples, a decrease of the Chl(*a + b*)/Car value was observed in comparison with the control (Table 4). This indicates oxidative stress in radish leaves and suggests the attempt of antioxidant defense carried out by the plant.

### Effect of ILs on MDA and H_2_O_2_ content

**Fig. 3:**
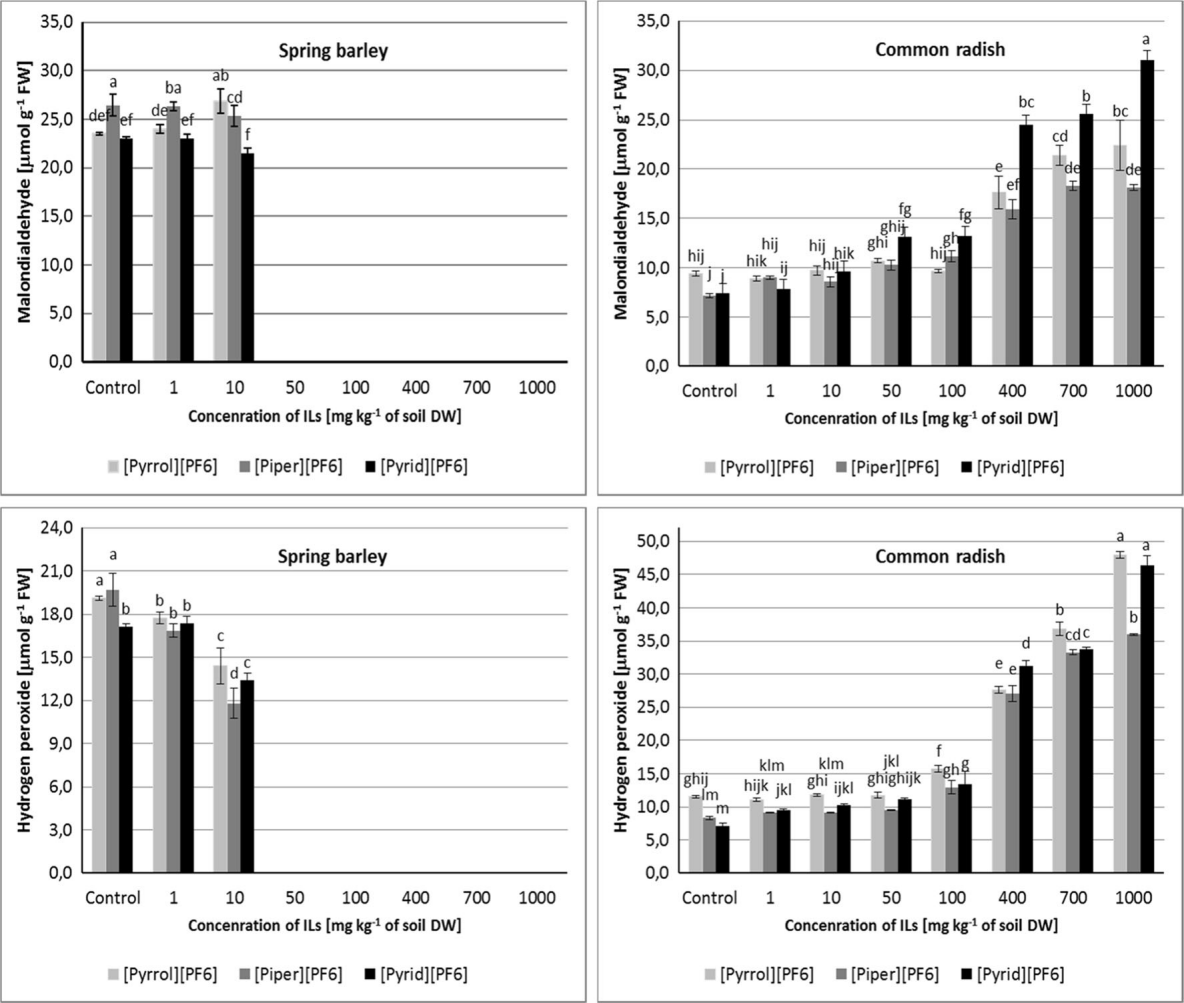
Effect of ILs on MDA (μmol g^−1^ FW) and H_2_O_2_ (μmol g^−1^ FW) content in spring barley seedlings and common radish leaves. Data are expressed as a mean ± SD of three replicates for each concentration. *Values denoted by the same letters* for each ILs do not differ statistically at *p <* 0.05

The peroxidation level of protein-lipid membranes in plant organisms is commonly determined on the basis of malondialdehyde content. Thus, the MDA content is one of the most important indices, always determined in the study of oxidative stress level in plants subjected to the influence of different stress factors (Liu et al. 2013, 2014; Rosalie et al. 2015; Radošević et al. 2015). In the present paper, no considerable changes of MDA content of spring barley seedlings were observed between controls and samples cultivated on the soil with the addition of ILs. However, a significant increase of MDA content in the common radish leaves, which at the highest IL concentrations averaged 250–400% in comparison to the control, was observed (Fig. 3). The greatest changes of MDA level were caused by the ionic liquid containing the aromatic cation [Pyrid][PF_6_].

In the available literature, studies can be found describing the effect of ILs on the biochemical and physiological changes in plants, reporting increase of MDA content in plant cells (Cvjetko Bubalo et al. 2014a; Liu et al. 2013, 2014, 2015a, 2016a, b; Biczak et al. 2016a). The authors explain that the observed tendencies in the changes of MDA level were caused by oxidative stress generated by high concentrations of ILs.

Another biomarker indicating oxidative stress in plants is the H_2_O_2_ accumulation in their cells. Hydrogen peroxide is the most stable chemical molecule of all ROS, characterized by the capability for a rapid penetration of cellular membranes. The increase of H_2_O_2_ content in plant cells is observed in the conditions of intensified superoxide anion radical detoxification and in the situation when enzymatic mechanisms of H_2_O_2_ detoxification fail in plants (Liu et al. 2013, 2015b; Sánchez-Rodríguez et al. 2010; Kumar et al. 2013; Demidchik 2015). Zhang et al. (2013), Liu et al. (2014), and Biczak (2016) found that H_2_O_2_ accumulation depends on the ILs’ concentration in the environment of plant vegetation.

The results revealed that extremely high accumulation of H_2_O_2_ was found in common radish cells, and the observed increase was positively correlated with the concentration of the used ionic liquid. The highest increase in the level of hydrogen peroxide was observed when high concentrations of [Pyrrol][PF_6_] and [Pyrid][PF_6_] were used (Fig. 3). On the contrary, H_2_O_2_ content in spring barley seedlings was decreased, which was the result of elevated activity of the enzymes responsible for decomposition of the ROS in comparison to the observed increased enzymatic activity of peroxidase and catalase in common radish leaves.

### Effect of ILs on antioxidant enzyme activities

Terrestrial plants have developed a system of antioxidant enzymes, which enables them to remove the excess ROS from the organism. The activities of SOD, CAT, POD, and glutathione reductase (GR) are correlated, since normally, the product of action of one of them constitutes an activator and substrate influencing another (Anjaneyulu et al. 2014; Gengmao et al. 2015; Sánchez-Rodríguez et al. 2010; Noqueirol et al. 2015).

In the scientific literature, a view prevails that the first line of defense against ROS is SOD, which decomposes the superoxide anion radical to H_2_O_2_ and O_2_. Therefore, the activity of the enzyme is always determined in scientific studies on the verification of the effect of oxidative stress on the biochemical and physiological changes in plants (Liu et al. 2013, 2015b, 2016a, b; Biczak 2016; Biczak et al. 2016a, b; Pawłowska and Biczak 2016; Zhang et al. 2013). Results of studies presented in the literature do not allow for reaching firm conclusions on the direction of changes in the activity of SOD in plants subject to the conditions of oxidative stress generated by ILs. The analysis of the present study results demonstrated that in spring barley seedlings, no statistically significant changes of the activity of superoxide dismutase between the control and plants cultivated in the soil with ILs’ addition were observed. The observed lack of changes in SOD activity in spring barley seedlings was because the analysis was performed only at the lowest concentrations of applied ILs (1 and 10 mg kg^−1^ of soil DW). On the contrary, a considerable increase in activity of the enzyme after the use of ILs was observed in common radish leaves. The observed increase of SOD activity occurred only to certain ILs’ concentration. In the highest concentrations of these salts used in the experiment, SOD activity decreased slightly; however, it always remained on the level higher than in the control (Table 5).

**Table 5:** Enzymatic activities of SOD (U mg^−1^ protein), CAT (U mg^−1^ protein min^−1^), and POD (U mg^−1^ protein min^−1^) in spring barley seedlings and common radish leaves treated with ILs

Concentration of ILs (mg kg^−1^ of soil DW)		The activity of enzymes		
		Superoxide dismutase (U mg^−1^ protein)	Catalase (U mg^−1^ protein min^−1^)	Peroxidase (U mg^−1^ protein min^−1^)
Spring barley				
[Pyrrol][PF_6_]	0	9.901 ± 0.943bc	0.0483 ± 0.0014cd	14.868 ± 0.212cd
	1	10.113 ± 0.168abc	0.0458 ± 0.0005c	16.115 ± 0.082c
	10	8.950 ± 0.411c	0.0591 ± 0.0020a	38.649 ± 2.813a
	50	–	–	–
	100	–	–	–
	400	–	–	–
	700	–	–	–
	1000	–	–	–
[Piper][PF_6_]	0	11.223 ± 0.998ab	0.0434 ± 0.0014ef	9.897 ± 0.360ef
	1	11.769 ± 0.213a	0.0448 ± 0.0014de	12.215 ± 0.175de
	10	11.781 ± 0.280a	0.0519 ± 0.0025b	26.976 ± 0.411b
	50	–	–	–
	100	–	–	–
	400	–	–	–
	700	–	–	–
	1000	–	–	–
[Pyrid][PF_6_]	0	11.174 ± 0.787ab	0.0286 ± 0.0016e	6.424 ± 0.100g
	1	11.373 ± 0.522ab	0.0293 ± 0.0016e	8.074 ± 0.288fg
	10	9.103 ± 0.225c	0.0448 ± 0.0013cd	13.118 ± 0.454d
	50	–	–	–
	100	–	–	–
	400	–	–	–
	700	–	–	–
	1000	–	–	–
Common radish				
[Pyrrol][PF_6_]	0	10.915 ± 0.085ghijk	0.0255 ± 0.0014efghi	0.897 ± 0.019kl
	1	11.415 ± 0.712fghij	0.0244 ± 0.001bghi	0.827 ± 0.085kl
	10	10.823 ± 0.455ghijk	0.0231 ± 0.0026ij	0.895 ± 0.005kl
	50	10.579 ± 0.207hijk	0.0281 ± 0.0023defgh	2.409 ± 0.056hi
	100	10.501 ± 0.398ijk	0.0320 ± 0.0306cd	2.739 ± 0.177gh
	400	19.271 ± 0.384bc	0.0370 ± 0.0009b	9.510 ± 0.227de
	700	17.931 ± 0.647cde	0.0334 ± 0.0008bc	9.901 ± 0.380cd
	1000	17.734 ± 0.175de	0.0333 ± 0.0003bc	10.521 ± 0.031c
[Piper][PF_6_]	0	12.197 ± 0.612fg	0.0164 ± 0.0001kl	1.161 ± 0.086jkl
	1	11.953 ± 0.379fgh	0.0148 ± 0.0010l	1.081 ± 0.167kl
	10	12.698 ± 0.490f	0.0147 ± 0.0014l	1.387 ± 0.059jk
	50	12.340 ± 0.068f	0.0241 ± 0.0014hi	1.911 ± 0.123ij
	100	12.548 ± 0.165f	0.0295 ± 0.0014cde	3.208 ± 0.083g
	400	22.380 ± 0.574a	0.0417 ± 0.0009a	12.551 ± 0.358b
	700	19.489 ± 0.800b	0.0283 ± 0.0009defg	14.211 ± 0.206a
	1000	18.654 ± 0.496bcd	0.0191 ± 0.0009jk	14.666 ± 0.370a
[Pyrid][PF_6_]	0	10.058 ± 0.263jk	0.0148 ± 0.0016l	0.588 ± 0.021l
	1	11.722 ± 0.721fghi	0.0142 ± 0.0005l	0.667 ± 0.039kl
	10	10.011 ± 0.050jk	0.0140 ± 0.0001l	0.710 ± 0.015kl
	50	9.732 ± 0.134k	0.0226 ± 0.0023ij	1.126 ± 0.079kl
	100	10.489 ± 0.387ijk	0.0254 ± 0.0014fghi	2.426 ± 0.094hi
	400	17.112 ± 0.515e	0.0279 ± 0.0016defgh	7.301 ± 0.102f
	700	17.264 ± 0.490de	0.0289 ± 0.0009def	7.860 ± 0.527f
	1000	17.670 ± 0.195de	0.0251 ± 0.0009fghi	8.808 ± 0.736e

Data are expressed as a mean ± SD of three replicates for each concentration. Values denoted by the same letters in the columns do not differ statistically at *p* < 0.05

Similar conclusions were drawn by other authors (Cvjetko Bubalo et al. 2014a; Liu et al. 2013, 2015a, b, 2016b; Biczak 2016) who proved that the increase of SOD activity determined at lower ILs’ concentrations indicates the defense of the plants against oxidative stress. On the contrary, high ionic liquid concentrations may lead to a significant damage of plant cells, which disables the introduction of antioxidant enzymes. The situation continues to reduce the possibility for antioxidant defense, eventually leading to the death of the cells and the entire plant organism.

H_2_O_2_ formed as a result of superoxide anion radical dismutation is digested by CAT and POD. The reaction of direct disproportionation of H_2_O_2_ to H_2_O and O_2_ is conducted by catalase. Despite the fact that some authors (Anjaneyulu et al. 2014; Chen et al. 2014) consider CAT as the basic enzyme responsible for the removal of H_2_O_2_ from plant cells, the data present in the scientific literature on the changes of activity of this enzyme are not sufficient to conclude the direction of such changes. Some authors (Cvjetko Bubalo et al. 2014a; Liu et al. 2014, 2016a, b; Pawłowska and Biczak 2016; Gengmao et al. 2015) demonstrated that CAT activity always increases under ILs’ induced oxidative stress. However, in some studies (Liu et al. 2014; Sánchez-Rodríguez et al. 2010), a considerable decrease of the enzyme’s activity was observed in plants subjected to oxidative stress. In spring barley seedlings, a slight increase of CAT activity was observed at the highest IL concentration (10 mg kg^−1^ of soil DW) (Table 5).

The changes of POD activity are considered to be the most reliable biomarker indicating the occurrence of oxidative stress in plants. Regardless of the cause for oxidative stress, the activity of the enzyme always increases under the excess of H_2_O_2_ (Liu et al. 2013; Biczak 2016; Biczak et al. 2016a, b; Anjaneyulu et al. 2014). According to Zhang et al. (2013), the observed increase of POD activity indicates stronger affinity of the enzyme to H_2_O_2_ than CAT. In the discussed study, a systematic increase of POD activity was determined in spring barley seedlings and common radish leaves, growing on the soil with increasing concentration of ILs. In the highest dosages of all applied ionic liquids, the observed increase of peroxidase activity in common radish leaves was very high and reached several hundred percent compared to the control (Table 5). However, some studies (Arias-Baldrich et al. 2015; Wang et al. 2009) have increasingly paid attention to the fact that the increase of POD activity is not entirely favorable for plant organisms. The increased POD activity may disable plant metabolism via removal of H_2_O_2_ molecules, which are responsible for the cellular signaling, and lead to the damage of chlorophyll particles.

## Conclusion

The analysis of the results on the influence of various ILs [Pyrrol][PF_6_], [Piper][PF_6_], and [Piryd][PF_6_] on the growth and development of spring barley and radish and the oxidative stress level allowed for drawing the following conclusions including

1. The used ILs, particularly in high concentrations, were clearly phytotoxic. Phytotoxicity depended on the plant species. Spring barley turned out to be more sensitive to the tested salts than common radish.
2. There were no significant differences in phytotoxicity of ILs with differentiated structure of cations for spring barley. In the case of common radish, modification of IL cation structure affected the size of the changes observed in selected parameters of phytotoxicity and oxidative stress. It was evident especially after the introduction of high concentrations of these salts into the soil.
3. All ILs caused a decrease of photosynthetic pigments’ content in spring barley seedlings and common radish leaves, which, as a consequence, caused the decrease of yield of both plants.
4. ILs led to oxidative stress, which was followed by the MDA accumulation in both plants and increase of H_2_O_2_ in common radish leaves.
5. In response to stress conditions, the plants activated a system of antioxidant enzymes—SOD, CAT, and POD. The most significant enhancement of activity was observed for peroxidase, which can be considered the basic biomarker of oxidative stress in spring barley and common radish.

## Electronic supplementary material

Suppl.Table 1 Effect of [Pyrrol][PF6], [Piper][PF6] and [Piryd][PF6] on the germination potential (GP) and germination rate (GR) of spring barley and common radish plants. Data are expressed as a mean ± SD of three replicates for each concentration. Values denoted by the same letters in the columns do not differ statistically at *p* < 0.05 (DOCX 20 kb)

Suppl.Fig. 1 Digital photographs of spring barley on the 14th day after introduction to the soil [Pyrrol][PF6], [Piper][PF6] and [Pyrid][PF6] (in mg kg-1 of soil DW) (PDF 238 kb)

Suppl.Fig. 2 Digital photographs of common radish on the 14th day after introduction to the soil [Pyrrol][PF6], [Piper][PF6] and [Pyrid][PF6] (in mg kg-1 of soil DW) (PDF 221 kb)
