# Supplementary material for: Role of cation structure in the phytotoxicity of ionic liquids: growth inhibition and oxidative stress in spring barley and common radish
Source: Environ Sci Pollut Res Int. 2017 Jun 22;24(22):18444–57. doi: 10.1007/s11356-017-9439-x (PMC5554276; doi:10.1007/s11356-017-9439-x)
Supplement: Supplementary file 1 — Effect of [Pyrrol][PF6], [Piper][PF6] and [Piryd][PF6] on the germination potential (GP) and germination rate (GR) of spring barley and common radish plants. Data are expressed as a mean ± SD of three replicates for each concentration. Values denoted by the same letters in the columns do not differ statistically at p < 0.05 (DOCX 20 kb) [file 11356_2017_9439_MOESM1_ESM.docx]

| ILs concentration (mg/kg of soil DW) | | Spring barley | | Common radish | |
| --- | --- | --- | --- | --- | --- |
|  |  | GP (%) | GR (%) | GP (%) | GR (%) |
| [Pyrrol][PF_6_] | 0 | 66.67 ± 2.89^ab^ | 83.33 ± 7.64^a^ | 75.00 ± 8.66^abc^ | 96.67 ± 2.89^ab^ |
|  | 1 | 60.00 ± 10.00^abc^ | 80.00 ± 13.23^ab^ | 81.67 ± 2.89^ab^ | 96.67 ± 5.77^ab^ |
|  | 10 | 73.33 ± 11.55^a^ | 81.67 ± 5.77^a^ | 86.67 ± 5.77^ab^ | 96.67 ± 2.89^ab^ |
|  | 50 | 58.33 ± 12.58^abc^ | 86.67 ± 2.89^a^ | 78.33 ± 5.77^ab^ | 93.33 ± 2.89^ab^ |
|  | 100 | 46.67 ± 2.89^bc^ | 75.00 ± 13.23^ab^ | 65.00 ± 13.23^bcd^ | 66.67 ± 2.89^c^ |
|  | 400 | 10.00 ± 8.66^e^ | 55.00 ± 5.00^bc^ | 68.33 ± 5.77^abcd^ | 68.33 ± 5.77^c^ |
|  | 700 | 0.00 ± 0.00 | 20.00 ± 5.00^dcf^ | 55.00 ± 5.00^cd^ | 66.67 ± 2.89^c^ |
|  | 1000 | 0.00 ± 0.00 | 0.00 ± 0.00 | 50.00 ± 5.00^d^ | 66.67 ± 2.89^c^ |
| [Piper][PF_6_] | 0 | 58.33 ± 7.64^abc^ | 88.33 ± 7.64^a^ | 75.00 ± 8.66^abc^ | 90.00 ± 8.66^ab^ |
|  | 1 | 61.67 ± 10.41^abc^ | 85.00 ± 5.00^a^ | 75.00 ± 5.00^abc^ | 86.67 ± 5.77^ab^ |
|  | 10 | 70.00 ± 13.23^ab^ | 91.67 ± 7.64^a^ | 83.33 ± 7.64^ab^ | 86.67 ± 7.64^ab^ |
|  | 50 | 50.00 ± 15.00^abc^ | 81.67 ± 12.58^a^ | 78.33 ± 5.77^ab^ | 86.67 ± 2.89^ab^ |
|  | 100 | 38.33 ± 2.89^cd^ | 78.33 ± 7.64^ab^ | 81.67 ± 2.89^ab^ | 86.67 ± 12.58^ab^ |
|  | 400 | 3.33 ± 2.89^e^ | 40.00 ± 10.00^cde^ | 85.00 ± 5.00^ab^ | 90.00 ± 10.00^ab^ |
|  | 700 | 0.00 ± 0.00 | 15.00 ± 8.66^ef^ | 76.67 ± 2.89^abc^ | 81.67 ± 2.89^abc^ |
|  | 1000 | 0.00 ± 0.00 | 1.67 ± 2.89^f^ | 68.33 ± 2.89^abcd^ | 78.33 ± 10.41^bc^ |
| [Pyrid][PF_6_] | 0 | 66.67 ± 10.41^ab^ | 90.00 ± 8.66^a^ | 90.00 ± 10.00^a^ | 91.67 ± 7.64^ab^ |
|  | 1 | 65.00 ± 5.00^ab^ | 73.33 ± 5.77^ab^ | 83.33 ± 5.77^ab^ | 85.00 ± 8.66^abc^ |
|  | 10 | 60.00 ± 5.00^abc^ | 73.33 ± 7.64^ab^ | 78.33 ± 7.64^ab^ | 85.00 ± 8.66^abc^ |
|  | 50 | 60.00 ± 8.66^abc^ | 76.67 ± 10.41^ab^ | 80.00 ± 8.66^ab^ | 88.33 ± 2.89^ab^ |
|  | 100 | 46.67 ± 7.64^bc^ | 83.33 ± 10.41^a^ | 81.67 ± 10.41^ab^ | 88.33 ± 5.77^ab^ |
|  | 400 | 13.33 ± 7.64^de^ | 41.67 ± 10.41^cd^ | 66.67 ± 5.77^bcd^ | 93.33 ± 5.77^ab^ |
|  | 700 | 0.00 ± 0.00 | 15.00 ± 8.66^ef^ | 73.33 ± 7.64^abc^ | 98.33 ± 2.89^a^ |
|  | 1000 | 0.00 ± 0.00 | 15.00 ± 5.00^ef^ | 68.33 ± 2.89^abcd^ | 91.67 ± 2.89^ab^ |

*GP – germination potential, GR – germination rate*
