# Supplementary figures and images for: Role of cation structure in the phytotoxicity of ionic liquids: growth inhibition and oxidative stress in spring barley and common radish
Source: Environ Sci Pollut Res Int. 2017 Jun 22;24(22):18444–57. doi: 10.1007/s11356-017-9439-x (PMC5554276; doi:10.1007/s11356-017-9439-x)

[Pyrrol][PF<sub>6</sub>]

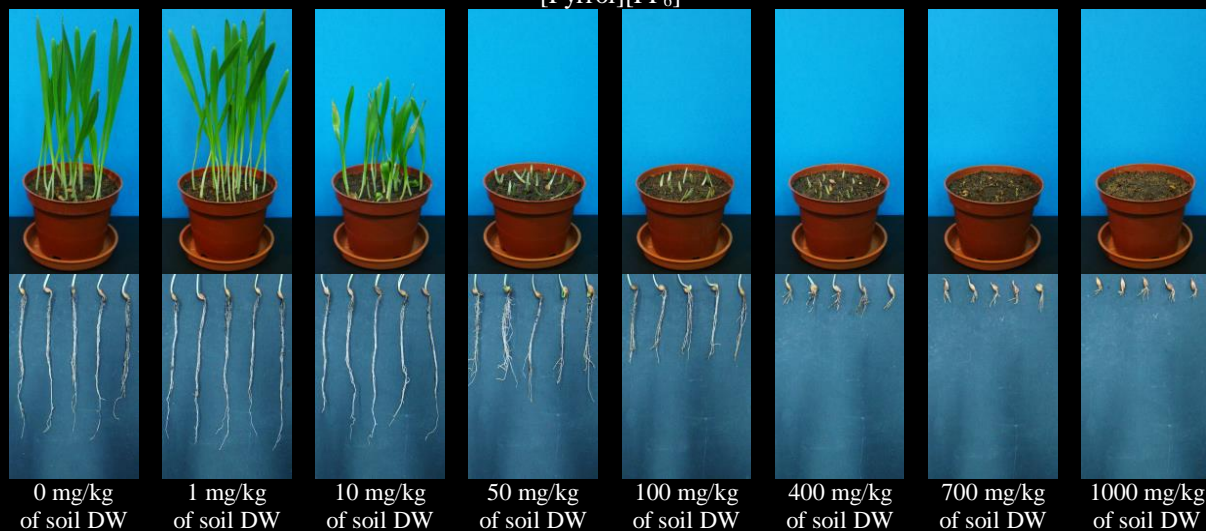

[Piper][PF<sub>6</sub>]

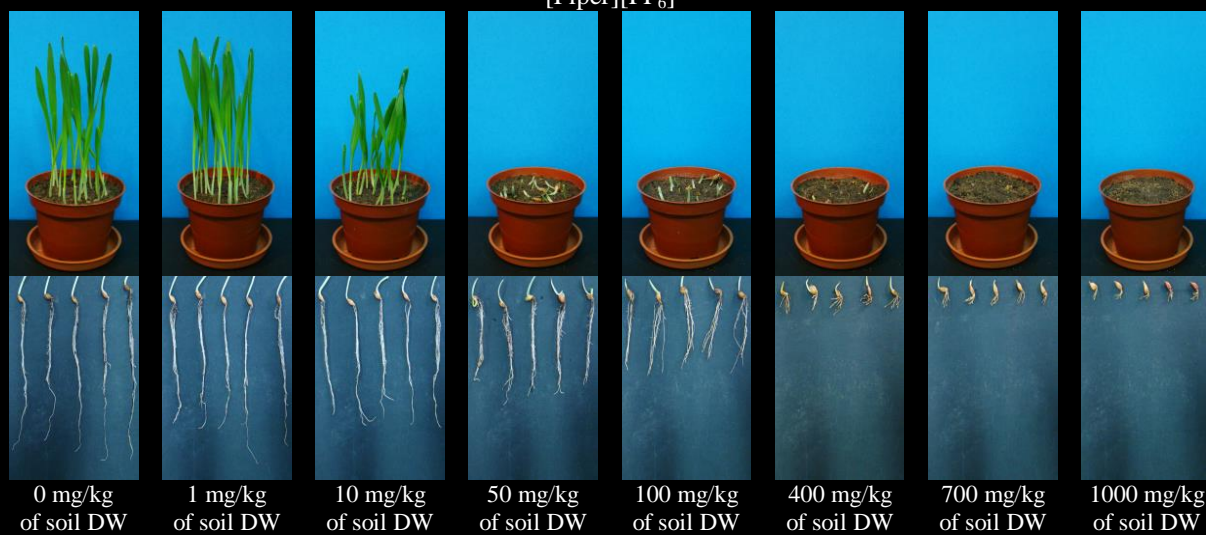

[Pyrid][PF<sub>6</sub>]

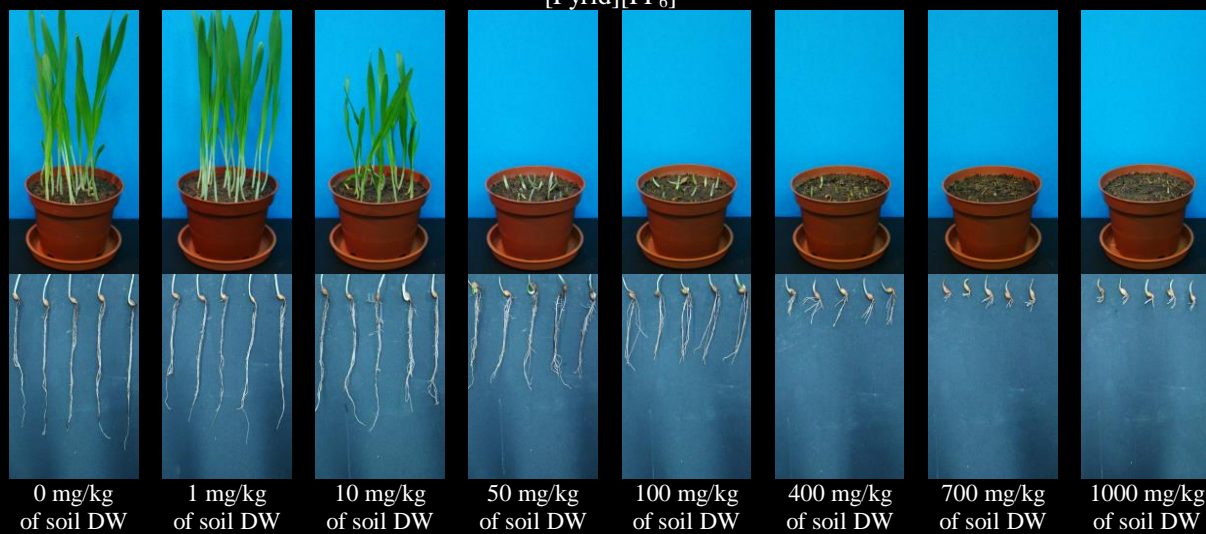

Supplement: Supplementary file 2 — Digital photographs of spring barley on the 14th day after introduction to the soil [Pyrrol][PF6], [Piper][PF6] and [Pyrid][PF6] (in mg kg-1 of soil DW) (PDF 238 kb) [file 11356_2017_9439_MOESM2_ESM.pdf]

[Pyrrol][PF<sub>6</sub>]

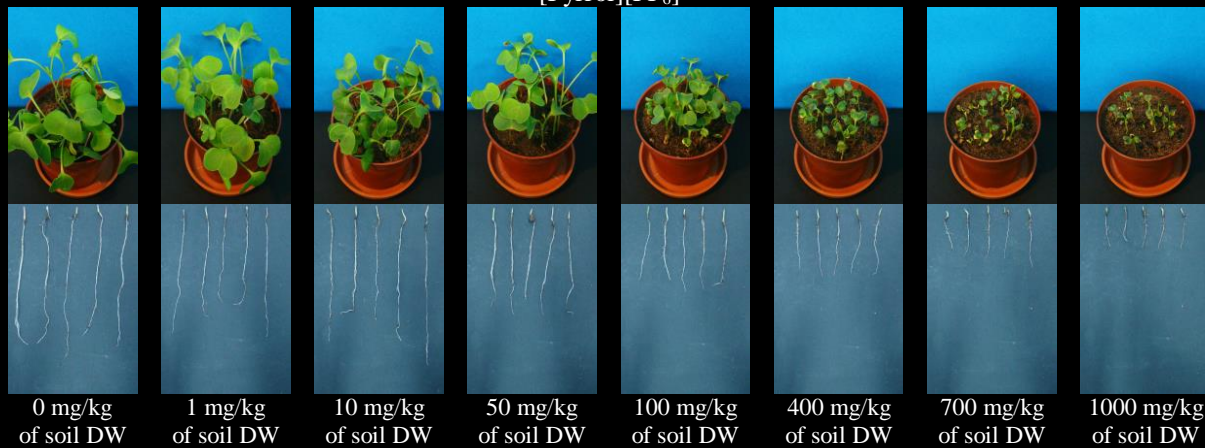

[Piper][PF<sub>6</sub>]

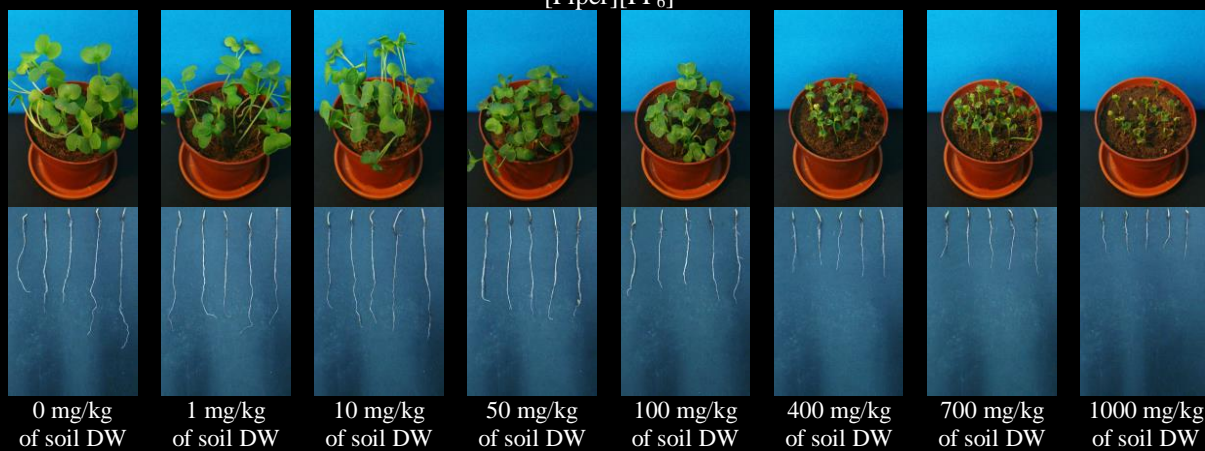

[Pyrid][PF<sub>6</sub>]

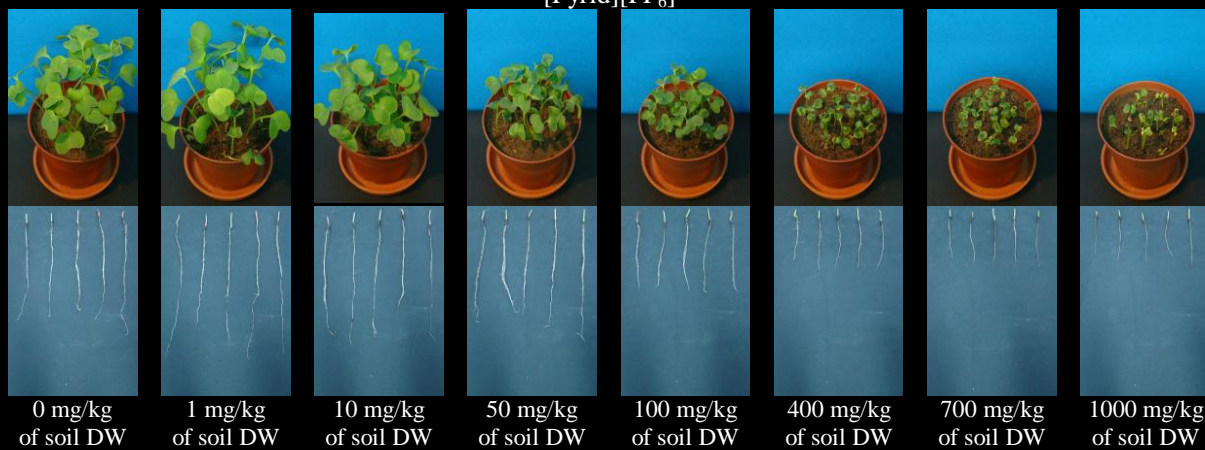

Supplement: Supplementary file 3 — Digital photographs of common radish on the 14th day after introduction to the soil [Pyrrol][PF6], [Piper][PF6] and [Pyrid][PF6] (in mg kg-1 of soil DW) (PDF 221 kb) [file 11356_2017_9439_MOESM3_ESM.pdf]
